# Ceramide homeostasis in hepatic lipid droplets

**DOI:** 10.1042/BST20253042

**Published:** 2025-05-01

**Authors:** Leobarda Robles-Martinez, Kylie H. Morin, Mariana Nikolova-Karakashian

**Affiliations:** 1 Department of Physiology, University of Kentucky, College of Medicine, Lexington, Kentucky 40502, U.S.A.

**Keywords:** biogenesis, ceramide, lipid droplets, mitochondria, steatosis

## Abstract

Almost all eukaryotic cells have the capacity to form lipid droplets (LDs) in conditions of excess energy. Traditionally thought to be just inert fat reservoirs, LDs have recently emerged as important metabolic regulators of cellular stress response that buffer excess free fats and protect cells from lipotoxicity. Ceramide is a bioactive lipid that accumulates in metabolic tissues during fat oversupply. Emerging evidence suggests that sphingolipids and sphingolipid-metabolizing enzymes are found in the LDs and affect LD biogenesis and functions. This article aims to summarize the evidence, delineate some plausible functions of ceramide in hepatic LD biogenesis, and illustrate some of the challenges in this novel field of research. We focus on the biogenesis of LDs in hepatocytes, the parenchymal cells of the liver, because non-alcoholic fatty liver disease is the quintessential manifestation of metabolic stress caused by fat oversupply.

## Introduction

The liver is the central hub co-ordinating the response of the organism to energy over- or undersupply. To that purpose, hepatocytes, the parenchymal cells of the liver, store excess fatty acids (FAs) in the form of inert triacylglycerol (TAG) in cytosolic lipid droplets (LDs). Almost all eukaryotic cells form LDs; however, only hepatocytes have the capacity to orchestrate a systemic response to supplement the peripheral organs with essential nutrients (lipids, glucose, ketone bodies, and amino acids) to sustain bodily functions when needed [1-4]. Recent studies have shown that LDs also sequester potentially lipotoxic metabolites such as free FAs, cholesterol, and ceramide [5-7] while fine-tuning the cellular response to metabolic stress. These newly discovered functions of LDs have been linked to the dynamic interactions between LDs and other subcellular organelles (e.g., mitochondria, endo-phagosomes, and lysosomes), allowing the controlled release/incorporation of lipids for maintenance of energy homeostasis while safeguarding against mitochondrial damage and lipotoxicity [6,8,9].

The LD hydrophobic core stores the excess free FAs in the form of TAG, diacylglycerol (DAG), and cholesterol esters. In the liver, the excess abundance of free FAs is caused by (i) elevated supply from the circulation due to obesity and (ii) up-regulation of *de novo* lipogenesis and/or suboptimal secretion of TAG in the form of VLDL, a condition known as lipodystrophy. The latter are typically the result of the onset of metabolic syndrome. Remarkably, hepatocytes could also form LD under conditions of nutrient deprivation, when autophagy is activated in order to sustain energy homeostasis. For conciseness, this article is focused on LDs in obesity-associated non-alcoholic fatty liver disease (NAFLD).

**Figure 1:**
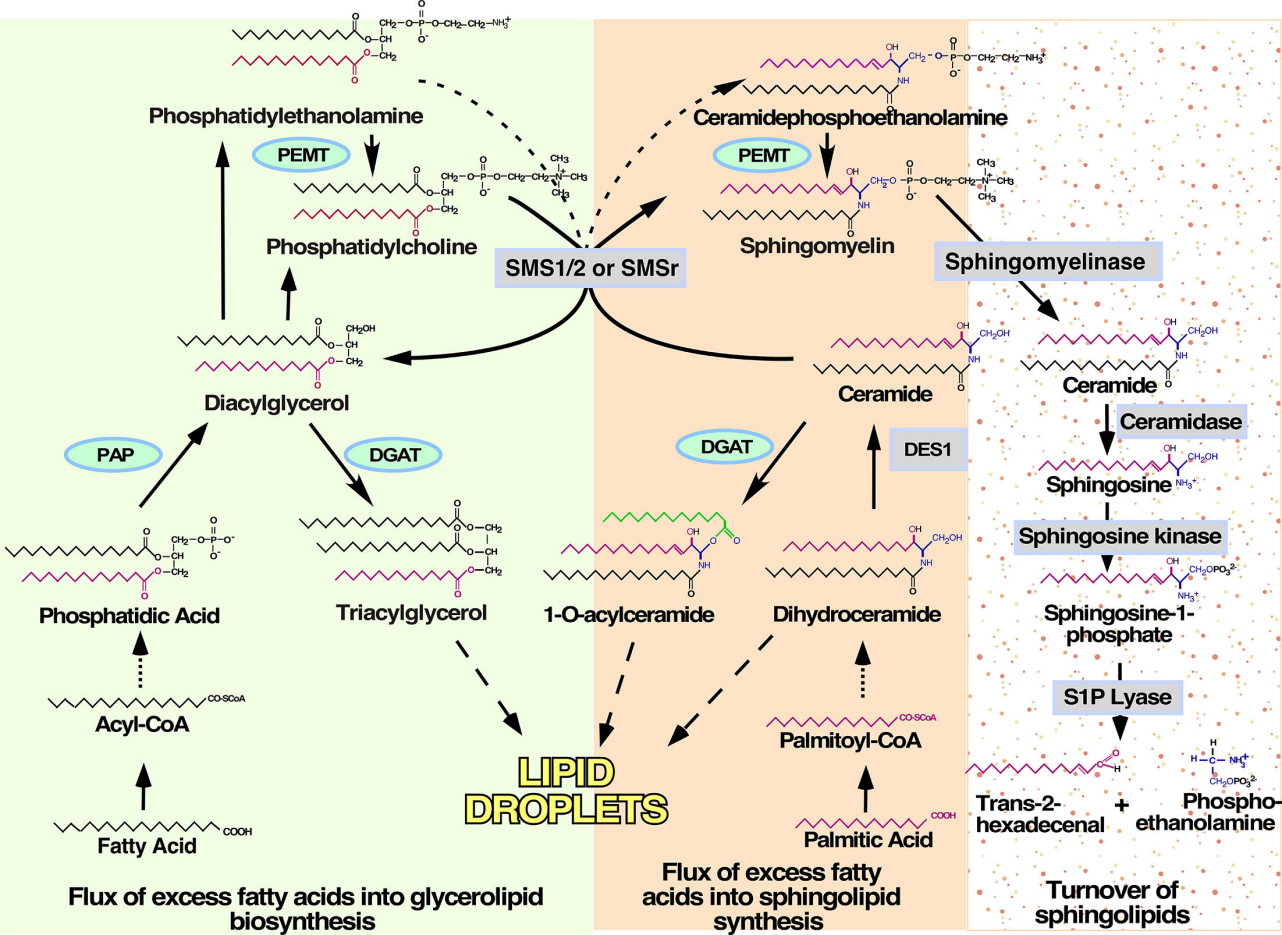
Schematic illustration of the flux of free fatty acid into glycerolipid and sphingolipid pathways leading to LD formation. In the liver, the excess abundance of free fatty acids is caused by (**i**) abnormal *de novo* lipogenesis, (ii) elevated supply from the circulation, or (iii) suboptimal secretion of TAG in the form of VLDL. The free fatty acids are incorporated to form glycerophospholipids, neutral lipids, or sphingolipids. The synthetic pathways for glycerophospholipids and TAG branch out at the level of DAG generated along the Kennedy pathways in the ER (green background). Excess fat could also enter the sphingolipid synthetic pathway (orange background). Notably, the rate-limiting step for *de novo* synthesis of the characteristic sphingoid base uses mostly C16:0 palmitic acid (in red color) but not fatty acids with other chain lengths of saturation. *De novo* synthesis of sphingolipids happens in the ER until ceramide generation. In the ER, excess ceramide could be acylated by DGAT similar to neutral DAG for storage in the LD. More complex sphingolipids, in particular SM, that are potential components of the phospholipid LD shell can be produced at different locations, based on known subcellular collation of respective enzymes. The major form of SM synthase, SMS1 is located in the Golgi apparatus and produces the majority of SM in the hepatocytes by transferring the phosphocholine headgroup of phosphatidylcholine to ceramide. A minor form, named SMS2, has been found in the plasma membrane to produce about 10% of hepatic SM via similar reaction. The only form that theoretically could produce SM in the ER is SMSr, which utilizes phosphatidylethanolamine as a substrate and generates transiently an SM analog, ceramide phosphoethanolamine that is methylated in a three-step methylation reaction to form SM. Turnover of sphingolipids is catalyzed by a distinct set of enzymes, namely sphingomyelinases, ceramidases, sphingosine kinases, and sphingosine 1 phosphate lyase, resulting in irreversible destruction of the sphingoid base that could not re-enter the fatty acid pool but has been found conjugated to some proteins and/or DNA. DAG, diacylglycerol; DES1, Dihydroceramide sphingolipid D4-desaturase 1; DGAT, diacylglycerol acyltransferase; LD, lipid droplet; SMS, sphingomyelin synthase; PAP, phosphatidic acid phosphatase; PEMT, phosphatidylethanolamine N-methyltransferase; S1P, Sphigosine-1-Phosphate; SM, sphingomyelin; TAG, triacylglycerol.

**Figure 2:**
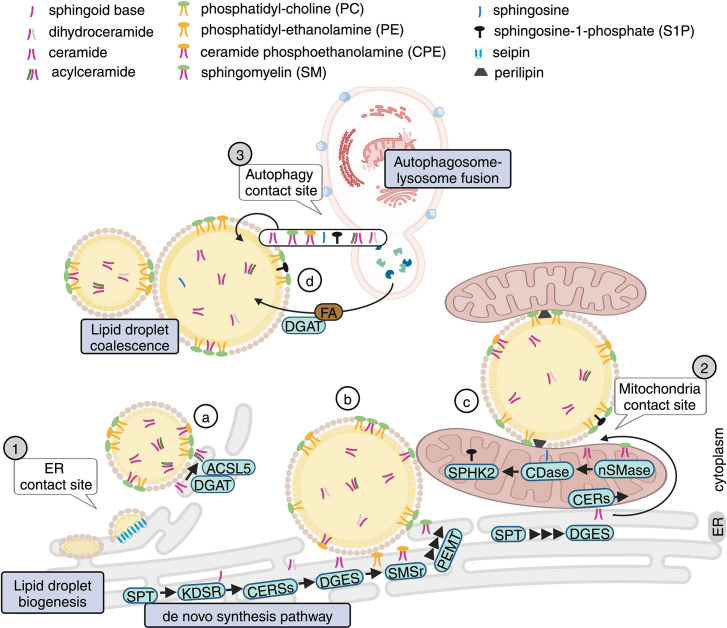
Origin of sphingolipids in LDs: organelles and enzymes The origin of sphingolipids in LD remains poorly understood. Based on current understanding of LD functions and subcellular location of specific enzymes in sphingolipid metabolism, the following possibilities have been illustrated: *Incorporation of sphingolipids during the initial formation of LD hydrophobic core*. (**a**) Ceramide can be O-acylated by diacylglycerol O-acyltransferases (DGATs) in co-ordination with long-chain fatty acid (FA) CoA ligase 5 (ACSL5) for FA synthesis, forming acylceramide, which is stored in LDs. Dihydroceramides could be incorporated in LDs during the initial budding of LD from the ER prior to the desaturation of ceramide by DES1 and their transfer to the Golgi apparatus. (**b**) Hypothetically, incorporation of SM in LD shell during the initial formation of LDs ER could happen via transfer of phosphoethanolamine headgroup to ceramide (catalyzed by SMSr) and followed by PEMT-mediated metylation of CPE to SM. *Remodeling of sphingolipid content of LD during LD expansion/degradation through LD:mitochondria contact sites (possibly energy-dependent*). Mitochondria and mitochondria-associated membranes (MAMs) contain several sphingolipid enzymes, including sphingomyelinase, ceramidase, and sphingosine kinase that may locally produce lipids for Mitochondria:LD transfer or may act on sphingolipids on the LD surface during LD:Mitochondria contact. *Incorporation of sphingolipids during coalescence of LD*. In the case of stress-induced LD formation, LD contact with autophagosome following autophagosomal lysosomal fusion may serve to transfer sphingolipids of lysosomal origin onto LD, similar to the process established for free FAs. Nutrient starvation activates the autophagy-mediated breakdown of membranous organelles leading to the accumulation of ceramide or dihydroceramide, and various saturated and unsaturated FAs. The release of FAs drive diacylglycerol acyltransferase 1 (DGAT1)-mediated LD biogenesis, while ceramide and other sphingolipids could be mobilized to LDs to buffer potential cytotoxic effects during autophagy. *In situ remodeling (not shown):* The discovery of sphingolipid metabolizing enzymes present at the LD surface suggests that sphingolipids species could be modified on ‘demand’ on LD surface. CPE, ceramide phosphoethanolamine; LD, lipid droplet; PEMT, PEMT, phosphatidylethanolamine N-methyltransferase; SM, sphingomyelin.

Several metabolic pathways underlie the conversion of excess free FAs to TAG for storage in LDs (Figure 1, green-shaded panel) [10,11]. The major form of AcylCoA synthase that is expressed in hepatocytes is ACSL5, and it catalyzes the conversion of FAs to their respective Acyl-CoAs destined for the formation of TAG for LD storage. Diacylglycerol acyltransferase 1 (DGAT1) preferentially channels exogenous FAs into TAG, whereas DGAT2 is more selective to saturated and monounsaturated FAs derived from *de novo* lipogenesis. According to the current paradigm, following DGAT1- and DGAT2-catalyzed acylation of DAGs to TAGs, LD hydrophobic core is formed in the ER, and when TAG levels reach 3.0–10.0  mol%, TAGs form lens structures at sites defined by specific regulated proteins and bud from the cytosolic face of the ER membrane (Figure 2) [12-14]. Once formed, LDs may expand further through fusion of two smaller droplets or as a result of *in situ* lipid synthesis on the LD surface. Coalescence of small LDs into a single larger droplet is facilitated by specific proteins like the CIDE family, which interact with phosphatidic acid on the LD surface and some LD scaffolding proteins. In turn, interactions of LDs with the enzymes involved in TAG synthesis, such as acyl-CoA synthetase, GPAT4, and DGAT2, allow for the expansion of preexisting LDs by *in situ* TAG synthesis [10]. Surprisingly, although the phospholipid monolayer must also expand with the concomitant addition of TAG to the LD core, no enzymes involved in the synthesis of phospholipids are present on LDs [15], and likely a transient association with the ER and/or protein-mediated transfer of phospholipids occurs during LD expansion. LD catabolism and degradation is initiated in response to stress and starvation. It is caused by co-ordinated catabolism of surface proteins, phospholipid membranes, and core neutral lipids. It is regulated by several LD proteins, such as PLIN2, adipose triglyceride lipase encoded by the patatin-like phospholipase domain-containing protein 2 (PNPLA2), and cholesterol esterase. Degradation of LD is also achieved during autophagy in a process called lipophagy. The latter is initiated in response to the activation of nutrient-sensing mechanisms, including sirtuin 1 (SIRT1) and AMPK, and involves budding of autophagosomes from LD or direct fusion of lysosomes with LD.

NAFLD is the quintessential manifestation of metabolic stress on hepatocytes induced by fat oversupply. Hepatic steatosis is the first and reversible stage of NAFLD and, if not managed, could lead to severe complications, including insulin resistance and type II diabetes, lipotoxicity, oxidative stress, failure to down-regulate *de novo* lipogenesis and gluconeogenesis [16-18]. Among all lipids that accumulate in the liver during steatosis, ceramides have been considered the most toxic due to ceramide effects on apoptosis, mitochondria and ER damage, and the onset of insulin resistance, among others [19-21]. The consequences of ceramide accumulation during NAFLD have been extensively studied with respect to ceramide’s capacity to modulate the onset and progression of steatosis upstream of LD formation and are associated with the function of ceramide as a bioactive second messenger [22-26]. Very little is known about the presence, metabolism, and functions of ceramide (and other sphingolipids) in the LD [27]. This article aims to summarize the evidence of novel roles of sphingolipids in hepatic LD biogenesis and illustrate some of the challenges in this novel field of research. For conciseness, we will focus on the biogenesis of LDs generated during diet-induced steatosis.

### Remarkable features of sphingolipid structure dictate the distinct role of ceramide in LDs

Sphingolipids, in contrast with TAGs, are an unlikely source of free FAs for the rapid acetyl-CoA production in the mitochondria during unmet energy demand. This is because the acyl chain (that varies in length between 14 and 26 carbon) forming the ceramide backbone is amid-linked to the sphingoid base, and only ceramidase, rather than a lipase, can catalyze the release of this one free FA (sphingolipid versus glycerolipid synthesis is illustrated in Figure 1). Additionally, the degradation of the amino-alcohol representing the characteristic sphingoid base requires phosphorylation (by sphingosine kinases 1/2) and a lyase to generate hexadecenal (a long-chain aldehyde) and phosphoethanolamine (Figure 1). These specifics in sphingolipid structure suggest that sphingolipid-rich LDs would be poor and slow energy reserves. One could put forward the hypothesis that the main role(s) of the sphingolipids accumulating in the LDs are (i) structural, (ii) regulatory, or (iii) protective, by sequestering potentially toxic sphingolipid precursors and/or metabolites.

Evidence from recent studies using lipidomic and proteomic approaches further indicates that LDs have a distinct sphingolipid composition that is different from that of other subcellular organelles. The sphingolipid composition of LDs appears to depend upon the cause of LD formation (obesity, fasting, LPS, autophagy) and the cell type, suggesting that selective pathways for incorporation of sphingolipids during the initial LD formation, as well as *in situ* remodeling, are involved [15,28-36]. The finding of several sphingolipid-metabolizing enzymes present in the LDs via unbiased proteomic analysis supports further such possibility [12,13,34,37-39].

### Origins of LD sphingolipids: enzymes and organelles

One of the first evidence that ceramides are present in the hydrophobic core came from work in Dr. Lina Obeid’ lab [14]. These studies indicated that LDs sequester O-acylated ceramide species. These newly identified ceramide metabolites were generated at the ER/LD interface following ACSL5-DGAT2-CerS6-dependent acylation of the hydroxyl group of *de novo* generated ceramide. The overexpression of ACSL5 leads to down-regulation of levels of ceramide supporting the notion that lipotoxic ceramide species that are produced abundantly during excess fat overload could be neutralized and sequestered in LD. Such capacity to store ceramides might be particularly relevant for the management of steatosis. This is because an accumulation of toxic ceramide following an elevated flux in the *de novo* pathway (via serine palmitoyl transferase) in the ER has been well documented to occur during NAFLD and has been implicated in the onset of insulin resistance, ER stress, and lipotoxiciity.

The published data on the levels of ceramide in hepatic LD are somewhat conflicting and point to the aforementioned dependence upon the inducers of LD formation. In a model of viral hepatitis- or oleate-induced steatosis in Huh7 cells, ceramide levels of isolated LDs were between 80% and 90% of all sphingolipids measured via targeted mass spectrometry that quantified ceramides, sphingomyelin (SM), and glucosylceramides [31]. A recent study from our group in a mouse model of obesity-induced steatosis that is currently being submitted for publication [40] confirmed that LDs are enriched in ceramides that were 10–12 times more abundant than SM. Furthermore, we also found that the LDs were rich in dihydroceramides species, particularly C20-dihydroceramide, that are rarely detectable in whole liver extracts (unpublished observations). One possible interpretation is that a distinct pool of ceramide that was produced *de novo* and escaped DES1-mediated introduction of 4,5-trans double bond was incorporated into the LDs. It should be noted that inhibition of DES1 has been shown to alleviate some obesity-associated complications, including NAFLD [41]; thus, one could not exclude the possibility that excess dihydroceramides produced in the ER during stimulation of lipogenesis are incorporated in LDs as another ‘measure’ to sequester potentially harmful sphingolipid metabolites from accumulating in cellular membranes. Ceramide profile of purified LDs has also been investigated in the case of alcoholic fatty liver disease by shotgun lipidomics. These analyses found that C16:0-Cer, C24:1-Cer, and C24:0-Cer were the most abundant ceramide species [36].

According to a recent comprehensive analysis of the lipid content of LDs from hepatocytes of mice fed high-fat diet, SM accounts for approximately 2.7 mol% of the total phospholipids [15]. In our study, the proportion of SM in LD was significantly less (<1.0% of total lipid phosphate of the LDs). Interestingly, a third study suggested that there are different pools of SM within the LDs, one smaller, dynamic pool with a shorter half-life, and another relatively inert pool with a half-life on the order of hours to days [42]. Albeit this was never tested in liver, the outcome indicates the possibility of *in situ* remodeling of sphingolipids in the LDs.

The origin of SM on LD surface is less clear. Previous studies have shown that in addition to ceramide, complex sphingolipids, mostly SM, accumulate in the liver during steatosis [43,44] However, having in mind that the addition of the charged phosphocholine group on ceramide to form SM happens in the Golgi apparatus, and, to a much smaller extent, at the plasma membrane, it seems unlikely that SM is incorporated during the initial budding of LDs from ER. SM could possibly be transferred during LD interactions with other organelles via membrane contact sites and/or fusion (Figure 2). Alternatively, SM synthases present at other organelles might convert LD ceramide on LD surface to SM upon transient interacting with the LDs or at membrane contact sites. It is also plausible that SMSr, the only member of the SM synthase family present in the ER, is involved. SMSr is highly homologous to SMS1 and SMS2, the two main SM synthases; however, it catalyzes the transfer of phosphoethanolamine head group from phosphatidylethanolamine to ceramide producing an analog of SM, ceramide phosphoethanolamine (CPE). CPE is then N-methylated to SM by phosphatidylethanolamine N-methyltransferase found at the ER and the mitochondria-associated membranes (MAM) [45,46]. The same enzyme has been shown to facilitate the generation of PC from PE. More importantly, *in situ* remodeling of PE to PC has been documented on LD surface and has been implicated in regulating binding of some peripheral LD proteins [47]. Therefore, such a possibility is an attractive and experimentally testable hypothesis to elucidate the origin of SM on the LD surface.

### Ceramide-metabolizing enzymes in the LDs

Little is known about the presence of enzymes linked to ceramide homeostasis at the LDs. In spite of the current advancements in proteomics, studies on identifying the sphingolipid-metabolizing enzymes that are associated and might be functional on LD surface have encountered several formidable challenges [48]. As many proteins are loosely associated with LDs, losses during isolation of the LDs are inevitable and difficult to account for. Furthermore, because LDs are very dynamic organelles, the hepatic LD proteome may change rapidly in response to feeding, fasting, circadian rhythmicity, to name a few [49]. Even within a single cell, LDs are not uniform in regard to their size, maturation, proteome, and lipidome, which imposes additional obstacles in interpreting experimental outcomes. Finally, LD ceramide homeostasis might be under the control of proteins that do not fulfill the criteria of LD proteins (LDPs, i.e., being enriched in LDs), as these proteins could be more abundant in other subcellular organelles and to associate with LDs only transiently.

In our recent studies on the role of neutral sphingomyelinase 2 (nSMase2) in obesity-associated NAFLD, we observed that the onset of the steatosis was associated with the accumulation of nSMase2 on the surface of the LD [40] (submitted). nSMase2 is one of four sphingomyelinases that can generate the bioactive ceramide via hydrolysis of SM and is considered a bona fide signaling enzyme [50]. Our earlier studies have identified nSMase2 as a major contributor to the onset of insulin resistance in the liver [51]. A combined approach of using indirect immunofluorescence, enzymatic activity assays, lipidomics, and various methods of LD purification for biochemical analyses leads to the conclusion that purified LDs contained functionally active nSMase2, providing the first experimental evidence that LD ceramide homeostasis can be regulated *in situ* (in preparation for publications). A recently published study employing un-biased analyses of liver proteome and LD subproteome has identified several sphingolipid-metabolizing enzymes in purified LD (Table 1) [39], although they did not reach the extent of enrichment in the LDs required for core LD proteins (>10-fold) but were close to the threshold for peripherally associated proteins (>2-fold). These ‘periphery’ LD proteins can be found in other cellular locations and/or regulated during NAFLD but are enriched in LDs to a lesser extent than the core components. Another study on the proteome of LDs isolated from three human subjects with steatosis reported LD-associated acid ceramidase in all three patients and ceramide synthase in one patient. However, it should be pointed out that the extent of LD enrichment with this protein fell below the cutoff (10-fold) used to define core LD proteins in the mouse model.

**Table 1:** Sphingolipid-metabolizing enzymes identified in hepatic LD from obese and non-obese mice as compared with whole liver lysates [39].

			Enrichment LD/whole liver for the respective diet	
Symbol	Accession	Name	Chow	HFD
Smpd1	gi|6,755,582	Sphingomyelin phosphodiesterse precursor	0.85	4.40
Smpd2	gi|6,678,031	Sphingomyelin phosphodiesterase 2	0.06	9.03
Smpd3	gi|10,946,902	Sphingomyelin phosphodiesterase 3	1	7.94
Smpd4	gi|257,196,247	Sphingomyelin phosphodiesterase 4 isoform 2	0.73	4.61
Sgpl1	gi|31,543,694	Sphingosine-1-phosphate lyase 1	0.08	10.86
Kdsr	gi|110,625,780	3-Ketodihydro-sphingosine reductase precursor	1	5.20
Sphk2	gi|42,544,000	Sphingosine kinase 2	2.91	5.91
Sgpp1	gi|13,507,712	Sphingosine-1-phosphate phosphatase 1	0.85	4.32
Asah2	gi|9,055,168	Neutral ceramidase	0.8	5.10
Asah1	gi|9,790,019	Acid ceramidase precursor	0.35	7.65
St3gal5	gi|78,126,157	Lactosylceramide alpha-2,3-sialyltransferase isoform a	1.14	27.02
Gba2	gi|240,120,073	Non-lysosomal glucosylceramidase	0.73	6.3
Gba	gi|6,679,955	Glucosylceramidase isoform 1	0.2	7.1
Pnpla2	gi|254,826,780	Patatin-like phospholipase domain-containing protein 2 isoform 1	83.18	53.93
Plin5	gi|116,292,166	Perilipin-5	42.07	25.81
Dgat1	gi|6,753,632	Diacylglycerol O-acyltransferase 1	0.08	10.37
Plin2	gi|116,235,489	Perilipin-2	136.77	116.90

Proteins involved in sphingolipid metabolism are present in purified hepatic LDs of mice fed high-fat diet for 18 weeks. Data are excerpts from Supplemental Table 7 in Liu et al. [39] and represent the enrichment factor for each protein in LD. The enrichment factor is the ratio of isobaric tag recovery for each protein in LD and total liver extract based on iTRAQ-MS/MS. The enrichment factors for known LD ‘core’ proteins, e.g. LDPs, PLIN5, PLIN1, and DGAT1, are included for comparison.

### Functional significance of maintaining ceramide homeostasis in the LDs

Our understanding of the consequences of the dynamic regulation of ceramide levels in LD is presently incomplete. Solid evidence supports the concept that LDs could sequester toxic ceramide in the presence of excess fat, thereby protecting the cells from liptoxicity. The mechanisms that control the release of these sequestered metabolites during LD degradation and what is the impact (if any) on cell functions, however, remain unclear and need further investigation. At least one study has provided experimental evidence that specific increases in LD ceramide correlated with increases in LD size and implied that increases in ceramide levels on LD surface may influence the fusion and/or ER budding of the LDs [31]. Due to the presence of a 4,5-trans double bond and an additional hydroxyl group at C3, ceramide has been well known to increase the order of acyl chains in lipid bilayers leading to self-aggregation, membrane invagination, and the formation of smaller droplets [52,53]. Our observations suggest [40] nSMase2-mediated turnover of LD-SM to ceramide limits the capacity of hepatic LDs to expand only following prolonged (17–19 weeks) dietary feeding, but not at earlier times (i.e., 10–12 weeks). Close proximity of nSMase2 to the core LD protein, PLN3 has also been reported in oleate-treated Jurkat T cells [54], emphasizing the need to address this novel role of SM turnover on LD surface. Clearly, future studies should aim at deciphering the mechanisms of dynamic controls of ceramide homeostasis in the LD in physiologically relevant system of fat oversupply and/or starvation, especially in the context of interactions of LDs with other cellular organelles.

It is also conceivable that exposing ceramide to the LD surface may affect the capacity of LDs to bind to any the nearly 350 different proteins found in the LDs [55-60]. The generation of ceramide within a lipid bilayer has been shown to enhance the propensity for inverted micellar structure (hexagonal II phase) that provides hydrophobic interaction sites for proteins [61]. Furthermore, a specific ceramide-binding motif has been identified in just one protein (the ceramide transfer protein, CERT1), and more than 20 different proteins have been reported to directly bind ceramide [62]. Finally, another intriguing possibility that only recently has been indicated is that interactions between LDs and mitochondria, which are essential for the maintenance of metabolic homeostasis in the cells, may occur in ceramide-dependent manner and have an impact on overall mitochondria bioenergetics. Recent studies have revealed that mitochondria associated with LDs exhibit distinct bioenergetic properties as compared with cytosolic, free mitochondria and that this differentiation is important for maintaining energy homeostasis during metabolic stress. Yet, the mechanisms that regulate the interactions between peripheral LD proteins and mitochondria are poorly understood.

## Conclusion

The advancement in state-of the-art lipidomic and proteomic approaches applied to well-characterized subcellular fractions has provided solid evidence that sphingolipids and sphingolipid-metabolizing enzymes are present and functional in LD. The key role that hepatic LDs play in lipid handling and in orchestrating the organism’ response to energy demand points to the necessity of a focused approach to understand the role of sphingolipids in LD biogenesis. The notion that LDs can sequester toxic ceramide species during *de novo* lipogenesis of LD particles seems well supported by early experimental data. SM and ceramide present on LD surface ostensibly could affect LD expansion, the interaction with subcellular compartments and with core LD proteins. However, we know very little about the enzymes that control ceramide homeostasis in LD, their role (if any) in LD biogenesis, and most importantly, the associations between metabolic stress and deregulation and these LD-associated sphingolipid-metabolizing enzymes. Studies aimed at deciphering the regulation and subcellular translocation of sphingolipid metabolic enzymes to the LD are seemingly feasible, especially in relatively well-understood disease models like obesity-associated NAFLD, where important keystones about the principles of LD biogenesis, sphingolipid homeostasis, and the nature of metabolic deregulation have been already established. A note of caution is warranted though, as LD biogenesis is rather dynamic, and the type and length of obesogenic diet has a significant impact on LD content and metabolic deregulation. Future experiments have to preferably account for lipid metabolism at subcellular levels and changes in metabolic state in a complex physiologically relevant model. Although such experiments are challenging and time-consuming, the rewards could be substantial: It is tempting to propose that maintaining ceramide homeostasis in LDs might function as a rheostat of fine-tuning the metabolic response of the cells to excess energy supply or the lack thereof. Being metabolically distinct from TAG, sphingolipids are resistant to well-orchestrated mechanisms for storing and degrading fat based on energy demand that governs TAG. Yet, in spite being a poor energy reserve, ceramide is tightly regulated during cellular metabolic distress posing yet another enigma for the sphingolipid biology.

**Perspectives**

- Importance of the field
  ‘Lipid droplets (LDs): Friends or foes?’” has become a recurring and yet unanswered question in biomedical research. With the improved technologies for LD purification, proteomics, and lipidomics, it become evident that ceramides and ceramide-metabolizing enzymes are present in LDs and might hold the key for understanding LD functions in human disease.
- Summary of current thinking
  As compared with glycerophospholipids and neutral lipids, sphingolipids are present in LD core and surface at miniscule amounts. Ceramide homeostasis in LD is regulated in the course of NAFLD by the activation of *de novo* synthesis in the ER and *in situ*, on the LD surface by neutral sphingomyelinase 2 (nSMase2). The incorporation of ceramide in the LD core might have a beneficial effect for the cells by preventing ceramide lipotoxicity; the presence of excessive active nSMase2 at the LD surface, however, is likely detrimental as it seems to limit the capacity of LD to expand and to interact with mitochondria.
- Future research
  Future research on sphingolipid homeostasis in LD has to employ plethora of technics simultaneously, including biochemical, biophysical, and cell imaging analyses to account for several formidable challenges: (i) many proteins are loosely associated with LDs and can be lost during LD isolation; (ii) the cellular LD proteome/lipidome may change rapidly in response to feeding, fasting, circadian rhythmicity; (iii) LDs are not uniform in regard to their size, maturation, proteome, and lipidome even within a single cell; (iv) LD ceramide homeostasis might be under the control of proteins that do not fulfill the criteria of lipid droplet proteins (LDPs, i.e., being enriched in LDs), as these proteins could be more abundant in other subcellular organelles and to associate with LDs only transiently.

## Abbreviations

CPE: Ceramide Phosphoethanolamine
DAG: Diacylglycerol
DES1: dihydroceramide sphingolipid D4-desaturase
DGAT: diacylglycerol O-acyltransferase
LD: Lipid Droplet
PAP: phosphatidic acid phosphatase
PC: Phosphatidylcholine
PE: Phosphatidylethanolamine
PEMT: phosphatidylethanolamine N-methyltransferase
SIP: sphigosine-1-phosphate
SM: Sphingomyelin
SMS: sphingomyelin synthase
TAG: Triacylglecerol
